# Supplementary material for: T-Cell Dynamics Predicts Prognosis of Patients with Hepatocellular Carcinoma Receiving Atezolizumab Plus Bevacizumab
Source: Int J Mol Sci. 2024 Oct 11;25(20):10958. doi: 10.3390/ijms252010958 (PMC11507274; doi:10.3390/ijms252010958)
Supplement: Supplementary file 1 [file ijms-25-10958-s001.zip › Figure S1.pdf]

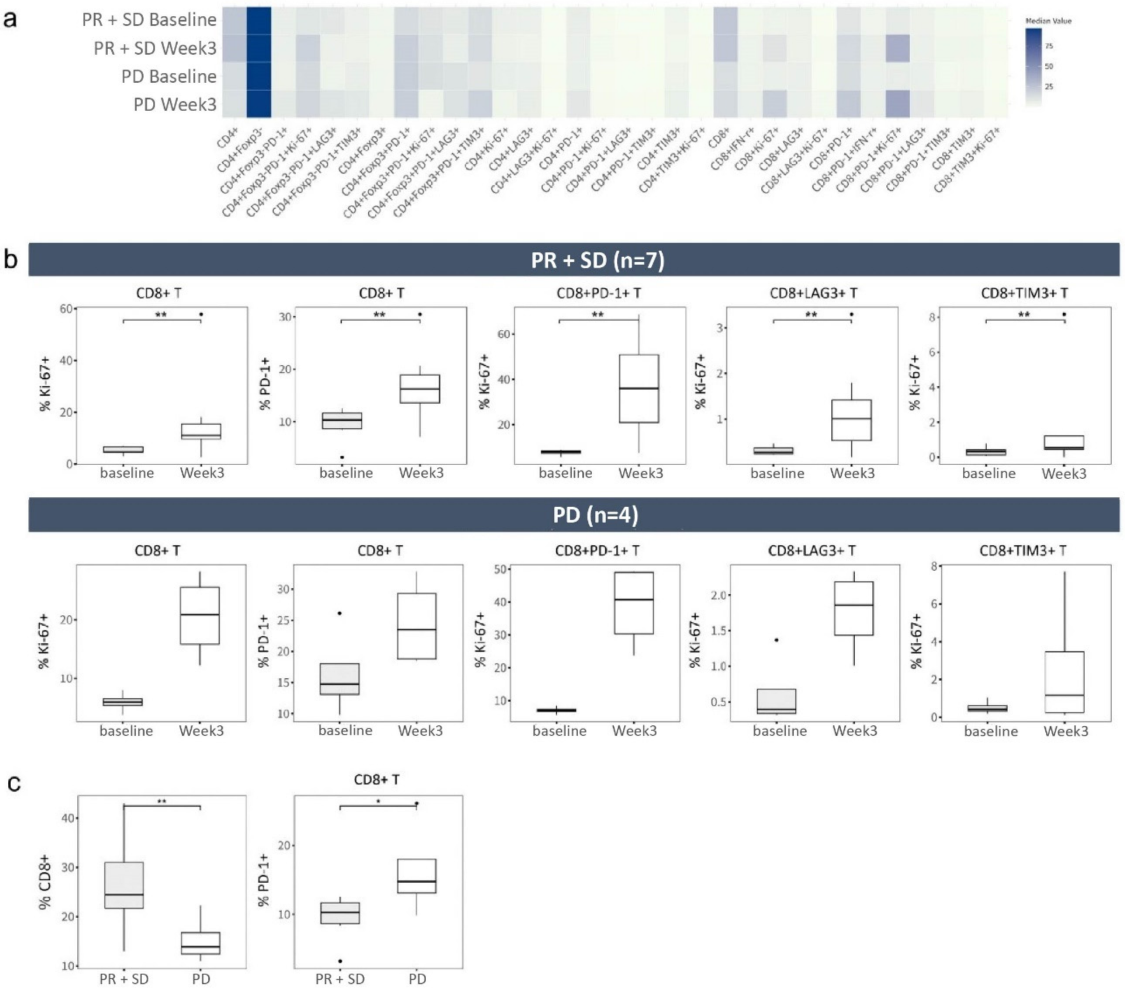


**Figure S1.** Immune response to atezolizumab plus bevacizumab in treatment-naïve patients (a) Heatmap of the median frequencies of 32 $CD4^{+}$ and $CD8^{+}$ T-cell phenotypes in PR+SD and PD at baseline and week 3 in 11 patients receiving first-line atezolizumab plus bevacizumab. (b) Box plots of the frequencies of T-cell phenotypes in PR+SD (n = 7) and PD (n = 4). $^{**}P < 0.05$, $^{*}P < 0.1$ according to Wilcoxon matched-pair signed-rank test. (c) Box plots of differences in the baseline frequencies of the indicated T-cell phenotypes in PR+SD and PD. $^{**}P < 0.05$, $^{*}P < 0.1$ according to Mann–Whitney U-test.
