# Supplementary material for: T-Cell Dynamics Predicts Prognosis of Patients with Hepatocellular Carcinoma Receiving Atezolizumab Plus Bevacizumab
Source: Int J Mol Sci. 2024 Oct 11;25(20):10958. doi: 10.3390/ijms252010958 (PMC11507274; doi:10.3390/ijms252010958)
Supplement: Supplementary file 1 [file ijms-25-10958-s001.zip › Table S1.pdf]

Table S1. List of reagents used in this study

Reagent name	Manufacturer	Catalog number
Anti-CD3 antibody	Thermo Fisher Scientific	11-0038-42
Anti-CD3 antibody	Thermo Fisher Scientific	58-0038-42
Anti-CD4 antibody	Thermo Fisher Scientific	12-0048-42
Anti-CD8 antibody	BD Biosciences	557760
Anti-CD279 (PD-1) antibody	Biolegend	329924
Anti-CD366 (TIM3) antibody	Thermo Fisher Scientific	56-3109-42
Anti-CD366 (TIM3) antibody	Thermo Fisher Scientific	11-3109-42
Anti-CD223 (LAG-3) antibody	Biolegend	369219
Anti-Foxp3 antibody	Thermo Fisher Scientific	58-4776-42
Anti-IFN-γ antibody	BD Biosciences	554701
Anti-Ki-67 antibody	Biolegend	350514
